# Influence of Processing Conditions on the Mechanical Behavior and Morphology of Injection Molded Poly(lactic-co-glycolic acid) 85:15

**DOI:** 10.1155/2017/6435076

**Published:** 2017-08-07

**Authors:** Liliane Pimenta de Melo, Gean Vitor Salmoria, Eduardo Alberto Fancello, Carlos Rodrigo de Mello Roesler

**Affiliations:** ^1^Biomechanical Engineering Laboratory (LEBm), University Hospital (HU), Federal University of Santa Catarina, 88040-900 Florianópolis, SC, Brazil; ^2^Laboratory of Innovation on Additive Manufacturing and Molding (NIMMA), Federal University of Santa Catarina, 88040-900 Florianópolis, SC, Brazil; ^3^GRANTE, Department of Mechanical Engineering, Federal University of Santa Catarina, 88040-900 Florianópolis, SC, Brazil

## Abstract

Two groups of PLGA specimens with different geometries (notched and unnotched) were injection molded under two melting temperatures and flow rates. The mechanical properties, morphology at the fracture surface, and residual stresses were evaluated for both processing conditions. The morphology of the fractured surfaces for both specimens showed brittle and smooth fracture features for the majority of the specimens. Fracture images of the notched specimens suggest that the surface failure mechanisms are different from the core failure. Polarized light techniques indicated birefringence in all specimens, especially those molded with lower temperature, which suggests residual stress due to rapid solidification. DSC analysis confirmed the existence of residual stress in all PLGA specimens. The specimens molded using the lower injection temperature and the low flow rate presented lower loss tangent values according to the DMA and higher residual stress as shown by DSC, and the photoelastic analysis showed extensive birefringence.

## 1. Introduction

Implants for medical applications using resorbable polymers derived from a class of aliphatic polyesters, polyhydroxy acids, are widely used for internal fracture fixation, wound closure, sutures, small vessel ligation, and drug delivery [1, 2]. During the injection molding process, polymeric materials undergo complex thermomechanical histories and significant changes in their rheological, mechanical, and thermochemical properties due to the large pressure variations, cooling times, mold geometry, and the manufacturing process [3–7]. The polymer's mechanical properties (apparent elastic modulus, maximum strength), morphology, crystallinity, and frozen layer thickness are also influenced by injection molding parameters, such as the melting process temperature, injection flow rate, holding pressure, mold temperature, and average bulk temperature [2, 8–11]. Poly(glycolide) and poly(L-lactide-co-glycolide), which are the synthetic copolymers of lactic acid (*α*-hydroxypropionic acid) and glycolic acid (hydroxyacetic acid), respectively, have good fiber-forming properties but their thermomechanical histories influence the ductility and degradability of the corresponding manufactured devices [12, 13].

The crystallinity and frozen layer thickness are controlled by the combined effect of the cooling rate and the stress fields imposed during the melting process [14–16]. Viana and collaborators [5] concluded that the thickness of the PLLA frozen layer increases with the stress level and decreases with temperature, while its degree of crystallinity increases with both shear stresses and temperature. On the other hand, Pantani et al. (2005) [17] indicated that the poly(acid lactide) frozen layer thickness increased when either the flow rate or the mold temperature decreased and that a correlation existed between the two parameters. Residual stresses and molecular orientations throughout a product provide important information about how that product will perform. Residual stresses are introduced by nearly all techniques used for polymer manufacturing and they can also be introduced by nonuniform flow, differential packing, or cooling. Therefore, an assessment of the mechanical behavior and structural characteristics of PLGA resulting from distinct injection molding parameters of absorbable polymers can provide valuable information.

This study provided an overview among processing conditions, morphology, and mechanical property relationship of injection molded PLGA. Two specimen groups with different geometries (notched and unnotched) were injection molded using two melting temperatures and flow rates (low and high). These choices generated four different processing conditions for both groups. For each processing condition, the mechanical properties (apparent elastic modulus, ultimate strength, elongation at failure, storage modulus, and loss tangent), morphology at fracture surface, and residual stress were evaluated.

## 2. Experimental 

### 2.1. Materials

Poly(lactic-co-glycolic acid) 85/15 granules, commercially available as Purasorb PLG 8531, purchased from Corbion Purac Biomaterials (Holland), were used in this study. The PLGA 85/15 average molecular weight of Mn = 224,271 g/mol and polydispersity of 1.87 were determined using Gel Permeation Chromatography (GPC) (Viscotek VE 2001, Viscotek detector TDA 302, USA, 2008). This copolymer has *T*_*g*_ of 57 ± 1°C, *T*_*m*_ = 140°C, and 3.04 dL/g of intrinsic viscosity (chloroform, 25°C, *c* = 0.1 g/dL).

### 2.2. Injection Molding Specimens

**Figure 1:**
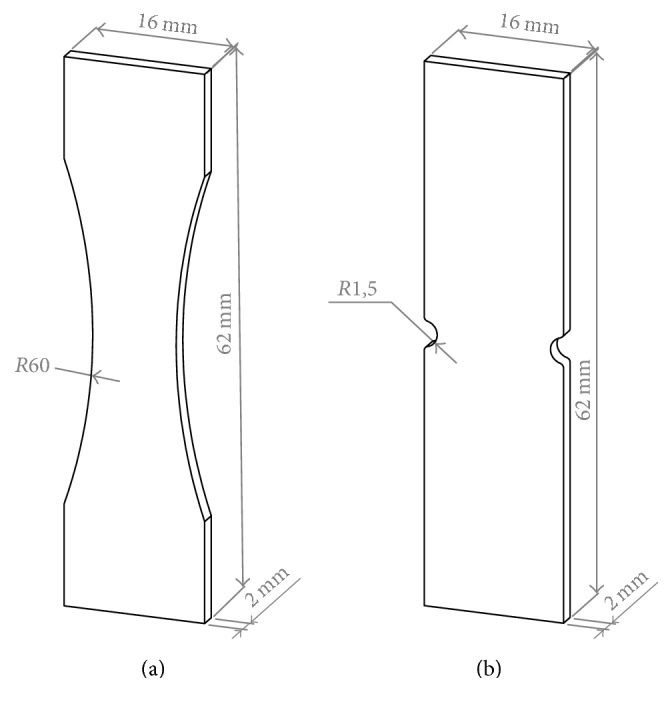
Illustration of the specimen's geometries used for injection molding: unnotched specimen (a) and notched specimen (b).

PLGA pellets were injection molded with an ARBURG 270S/250-70 machine into two groups of specimen geometries, notched and unnotched, adapted from ASTM D1822 type S and ASTM D638 type V. Both groups had a rectangular format of 62 × 16 mm of length and a cross section of 10 × 2 mm. Notched specimens had a 1.5 mm notch radius (stress concentration factor of 2.4), while unnotched specimens had a narrower section with a radius of 60 mm (see Figure 1).

Two (low and high) melt injection temperatures and two injection (low and high) flow rates were investigated, generating four injection conditions shown in Table 1.

The other processing parameters had the following fixed values: mold temperature: 25°C, injection pressure: 1500 MPa, holding pressure: 25 MPa, injection time: 2 s, cooling time: 90 s, and screw speed: 100 rpm.

### 2.3. Mechanical Characterization

#### 2.3.1. Tensile Test

The two different specimens were tested in an EMIC testing machine, model DL-3000, in the tensile mode as per ISO 527-1. The elongation of the specimens was measured using an extensometer, Instron/EMIC 2630-107. The tests were performed using a moving grip speed of 1 mm min^−1^ at a controlled room temperature of 23°C. Six specimens (*n* = 6) for each condition for each group were tested. The mechanical properties investigated were the apparent elastic modulus (taken as the initial slope of the engineering stress-strain curve) *E*, ultimate strength (maximum stress value of the engineering stress-strain curve) *σ*_*u*_, and engineering strain at failure *ɛ*_*f*_.

#### 2.3.2. Dynamical Mechanical Analysis

A DMA-Q800 analyzer (TA Instruments) with a single cantilever clamp was used for the viscoelastic tests. Dynamic mechanical analysis (DMA) provided the storage modulus *E*′ and tan⁡*δ* values at a frequency of 1 Hz within the temperature range of 30°C to 120°C using a heating rate of 3°C/min and a transversal displacement amplitude of 0.3% of the effective length of the specimen.

### 2.4. Scanning Electronic Microscopy

Scanning Electronic Microscopy (SEM) analysis was used to evaluate the fractured surface of the PLGA (85/15) specimens submitted to the tensile test and also to observe the frozen layer thickness and other morphological characteristics such as molecular orientation of shear force caused by molding injection. The analysis was conducted on all conditions of injection molding for the two groups of specimens.

In order to obtain good quality PLGA images, the specimens were fixed to a support with a double-sided carbon tape. For electronic conductivity, the specimens were covered with a thin layer of gold in a sputter model D2 Diode Sputtering System, manufactured by ISI (International Scientific Instruments). The fractured surfaces and thicknesses were observed using a JEOL JSM-6390LV (FEI Company, Japan) scanning electron microscope with an accelerating voltage of 5 kV.

### 2.5. Differential Scanning Calorimetry and Residual Stress Analysis

Differential scanning calorimetry (DSC) was used to determine the thermal transitions and residual stress enthalpy of the injection molded PLGA specimens in a Shimadzu DSC-6000 with nitrogen atmosphere (19 cm^3^ m^−1^), using aluminum oxide as standard. The heating rate was from 10°C to 250°C at 10°C m^−1^, using an average sample weight of 7 mg taken from the central region of the molded specimens. The residual stresses of the manufactured specimens were also evaluated by the polarized light technique using a polariscope with polarizing and quarter-wave lenses of 250 mm diameters, following the ASTM D4093 [18].

### 2.6. Data Analysis

Analysis of variance (ANOVA) was performed considering statistical significance set at 0.05; the *p* value was investigated for significance of the factors among injection molding conditions. All data are reported as mean ± standard deviation.

## 3. Results and Discussion

The processing conditions were systematically varied following a DOE array involving notch presence on geometry, melt temperature, and flow rate. Mechanical properties, as apparent elastic modulus, ultimate strength, elongation at failure, storage modulus, and loss tangent, were estimated by tensile test and DMA. The two morphological parameters, morphology at fracture surface and residual stress, were interpreted by the thermomechanical parameters. ANOVA was performed to measure statistically the significant response. The relationships between the morphology and mechanical properties were then established.

### 3.1. Tensile Test

**Figure 2:**
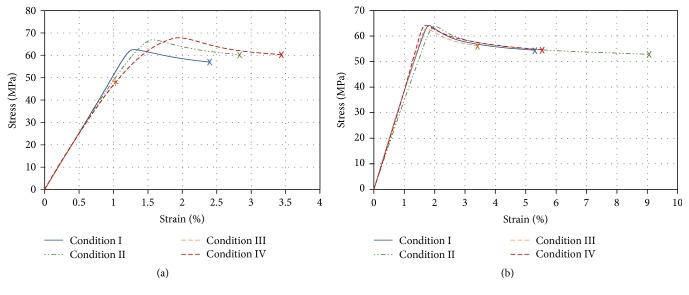
Stress-strain curves (means) of notched (a) and unnotched (b) specimens of injection molded PLGA.

Figure 2 shows the curves for stress versus strain for the notched and unnotched molded specimens using low and high values of melt injection temperatures and the two injection flow rates.

Table 2 contains the average values of *E*, *σ*_*u*_, and *ε*_*f*_ at each injection condition for both notched and unnotched groups. Similar mechanical properties were found for the injected PLGA specimens that were molded under different processing parameters (notched or unnotched samples), as shown in Table 2. But there is an evident difference between notched and unnotched samples relative to the apparent elastic modulus *E* mechanical property. The apparent elastic modulus *E* and ultimate strength *σ*_*u*_ show low sensitivity to the injection conditions for both the notched and the unnotched groups of specimens. However, there was a high standard deviation found for *σ*_*u*_ in the notched group injected under Condition III (see Table 2).

This low sensitivity reveals a certain level of material toughness. In order to achieve nearly the same value of *σ*_*u*_ for both geometries, the material localized near the notch valley seems to allow plastic deformation during loading to finally reach an almost constant stress distribution prior to the occurrence of a complete cross section (plastic) collapse. On the other hand, it is important to note that specimens, even those injected under the same conditions, showed different macroscopic behaviors at failure. While some showed a clear necking formation, others fractured without this formation. This observation is consistent with the large standard deviation found for *ε*_*f*_.

Sensitivity to the injection conditions showed that strains at failure presented slightly higher mean values for Conditions II and IV (low injection flow rates) than for Conditions I and III (high injection flow rates), for both notched and unnotched groups.

### 3.2. Scanning Electronic Microscopy

**Figure 3:**
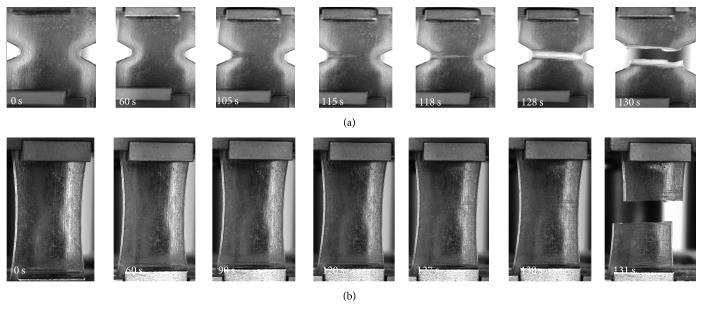
Sequence of images of tensile test for notched (a) and unnotched (b) specimens injected under Condition I.

Figure 3 presents a sequence of images that illustrate the progressive localization (necking) of strains prior to total failure of one of the notched specimens injected under Condition I. In these pictures, the capacity of the material to withstand plastic strain is macroscopically visible, as was already mentioned when discussing ultimate strength *σ*_*u*_.

**Figure 4:**
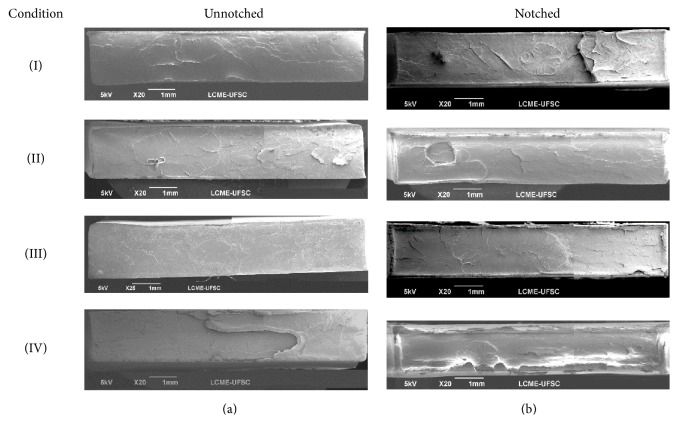
SEM of fractured surface unnotched (a) and notched (b) PLGA specimens molded under different processing conditions.

Unnotched specimens, as Figure 3 shows, present elongation along the specimen with longitudinal spread failure after 60 s, while notched specimens present stretching in the center region due to the stress concentrator (e.g., notch). Although there were different behaviors, the brittle feature of the material occurs in both geometries (see Figure 4).

It is worth emphasizing once more that a different macroscopic behavior at failure was observed even in specimens injected under the same condition; while some specimens presented clear necking formation, others failed without this formation showing flatter fracture surfaces (Figure 3).

SEM images of fractured surfaces are shown in Figure 4. These images are representative of those samples that did not show a clear necking formation. In these figures, it is possible to see flat fractured surfaces with some evidence of localized plastic flow, mainly in the notched specimens. The plastic fractures along the borders of the notched specimens are clear. This can be related to a different behavior between the core and borders and is due to the existence of the frozen layer. The existence of a thicker frozen layer in this group of specimens seems to be consistent with the fact that thicker frozen layers are related to higher flow rates as seen in the notched region of this group.

### 3.3. Dynamical Mechanical Analysis

**Figure 5:**
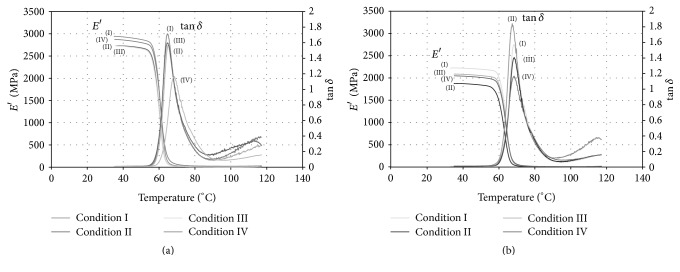
Storage modulus and loss tangent as a function of temperature for PLGA specimen injection molded under different conditions (I, II, III, and IV). Curve for unnotched (a) and notched (b) specimens.

Figure 5 shows representative curves of storage modulus *E*′ and tan⁡*δ* (tan⁡*δ* = *E*′′/*E*′) as functions of temperature for notched and unnotched specimens.

Sensitivity of the storage modulus to the injection conditions did not show a clear trend in the notched and unnotched specimens. Remember that due to the differences in geometry the storage moduli of the notched and unnotched specimens are not comparable. The only visible response is the lowest value of the loss tangent (tan⁡*δ*) reached by Condition IV (lower temperature and flow rate) for both notched and unnotched geometries, characterizing lower dissipation due to viscous micromechanisms.

Moreover, DMA was carried out to characterize the resulting copolymer in the four conditions. As shown in Figure 5, one obvious transition behavior was observed, designated as relaxation. It is well known that the glass transition temperature (*T*_*g*_) of a polymer can be determined by relaxation, as it is usually related to the segment movements in the noncrystalline area. This behavior, associated with broad peaks, nonexisting in a second heating scan, is characteristic of an incomplete crosslink process of copolymers formulations. In fact, tan⁡*δ* also indicates the composite damping capacity, which has a maximum value at the amorphous transition. As a PLGA copolymer, a possible reason why the curves went up above 100°C is the presence of residual monomers.

### 3.4. Differential Scanning Calorimetry and Residual Stress Analysis

**Figure 6:**
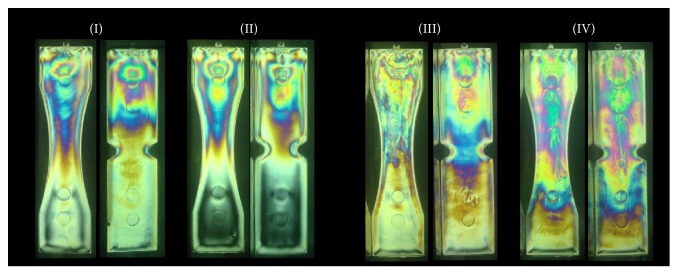
Photoelastic fringes of notched and unnotched specimens for Conditions I, II, III, and IV.

The injection molding of transparent polymers can induce a peculiar stress field that is clearly detected by photoelastic stress analysis [18, 19]. In Figure 6, the isochromatic maps for notched and unnotched specimens are shown. Notably, the isochromatic fringes have an asymmetric pattern distribution. This means that injection molding imposed an asymmetric thermomechanical environment onto the injected polymer that is related to the concentration of residual stresses near the injection gate.

The stress concentration decreases uniformly on the opposite side of the specimen at a different rate for each molding condition. The residual stresses arise during the filling and the packing processes. The wide distribution of residual stresses present in the specimens molded with the lower temperature is probably due to the nonuniform shear stress during cavity filling and rapid solidification. On the other hand, the concentrated residual stresses near the gate present in the specimens molded with the higher temperature are due to the compressive force caused by the holding pressure during the slower solidification.

In terms of stress shielding, Condition IV is the most propulsive to show a different behavior, since Condition 4 presents the lower temperature and lower flow rate. Then, the mold filling during injection molding along the specimen exhibited different characteristics due to the high shear stress between the polymer and the wall of the mold. The shear stress causes different lines of residual stress between notched and unnotched specimens.

**Figure 7:**
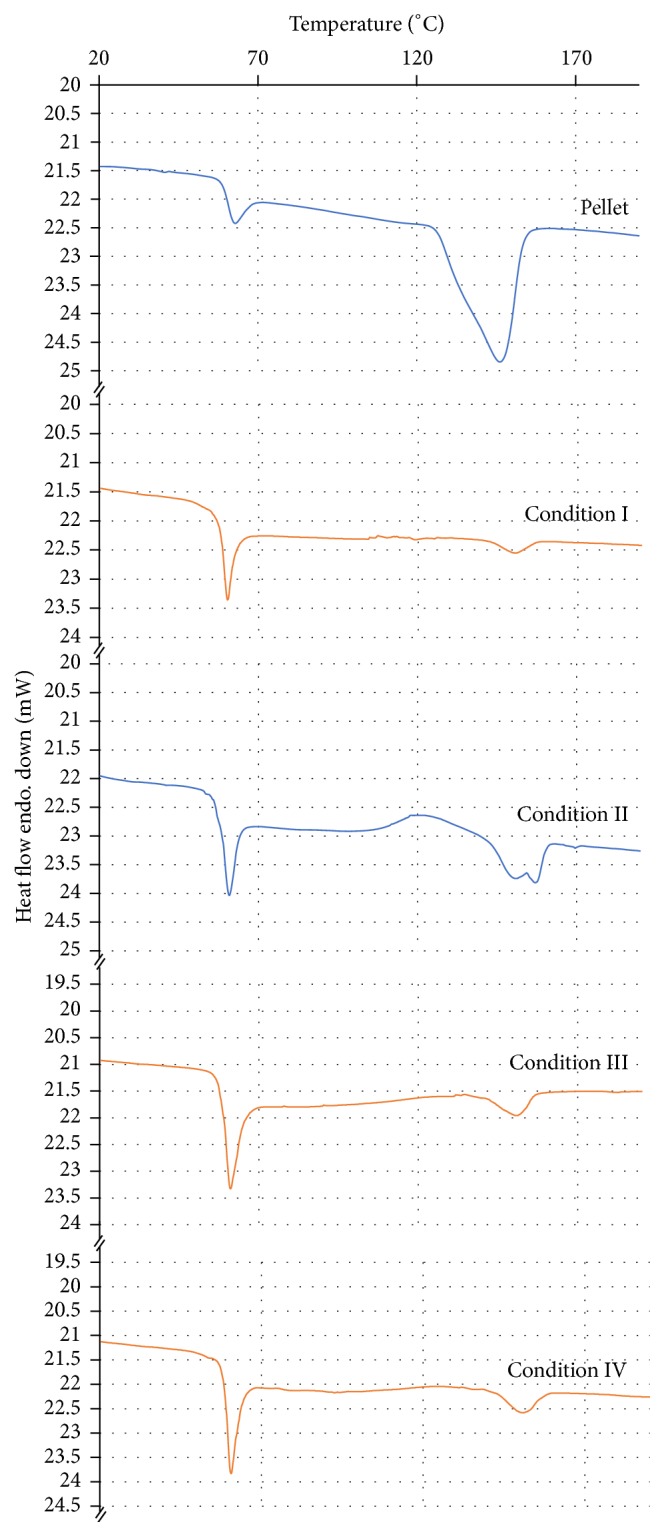
Calorimetry curves for PLGA pellets and molded specimens under different process conditions (I, II, III, and IV).

DSC curves (Figure 7) show the transitions for the PLGA pellets and for the PLGA molded specimens under different conditions. The molded specimen curves present a clear endothermic peak together with the glass transition related to the stress relaxation enthalpy of PLGA [20]. The residual stress was determined by measuring this relaxation enthalpy peak area at glass transition and is presented in Table 3. The relaxation enthalpy values were higher for the notched specimens molded under Conditions III and IV; that is, the lower temperature resulted in higher residual stresses at the center of the specimens in the notched region.

The DSC curves evidence the difference in the endothermic peaks and the presence of crystallinity only for Condition II. For this condition, a shoulder indicating two melting temperatures is present, related to PGA (15%) and PLA (85%) fractions, respectively, even though this value is very close to the detected *T*_*m*_.

Moreover, it is possible for residual stress to be reduced or eliminated with heat treatment to avoid influencing mechanical properties and fracture surface morphology of PLGA specimens. The present DSC curves are about the processed specimens without any treatment to show the results of the injection molding process. Heat treatment after the manufacturing of the devices in medical applications could possibly cause degradation of the material.

## 4. Conclusion

Similar mechanical properties were found for the injected PLGA specimens that were molded under different processing parameters. The melt temperature can influence the injection molded device and can be influenced by other parameters of injection molding. Flow rate is strongly associated with the shear rate and therefore has effects on melt temperature, molecular orientation, strains at failure, and residual stresses. The morphology of fractured surfaces of the notched and unnotched specimens showed flat and smooth fractures for the majority of the specimens. The macroscopic mechanical behavior of the injected specimens presented low notch sensitivity, suggesting the existence of a certain level of material toughness. The strains at failure presented slightly higher mean values for Conditions II and IV (low injection flow rate) than for Conditions I and III (high injection flow rate), for both notched and unnotched specimens. There were localized deformations near the specimen surface different from the core region. This can be related to the orientation of the skin layer, especially in the notched specimens. Polarized light techniques indicated birefringence throughout all specimens, especially in those molded under lower temperature, which suggests residual stress due to rapid solidification. DSC analysis confirmed the existence of residual stress in all PLGA specimens. The specimens molded using the lower injection temperature and lower flow rate (Condition IV) presented lower loss tangent values according to DMA and higher residual stress as shown by DSC, and photoelastic analysis demonstrated extensive birefringence along the specimen. Molecular restriction in the chain rotation and conformation due to the thick oriented skin layer can explain the less viscous behavior observed.

## Figures and Tables

**Table 1:** Injection molding conditions for notched and unnotched PLGA specimens.

Injection condition	*T* (°C)	*Q* (cm^3^ s^−1^)
I	240	25
II	240	10
III	210	25
IV	210	10

**Table 2:** Mechanical properties of notched and unnotched PLGA specimens injected with different molding conditions.

	Injection condition	*T*_inj_ (°C)	*Q*_inj_ (cm^3^ s^−1^)	*E* (GPa)	*σ*_*u*_ (MPa)	*ε*_*f*_ (%)
Notched	I	240	25	5.6 ± 0.4	63.1 ± 1.2	2.7 ± 0.3
	II	240	10	4.8 ± 0.2	65.5 ± 1.4	3.3 ± 0.5
	III	210	25	4.8 ± 0.4	54.0 ± 11.0	1.9 ± 1.0
	IV	210	10	4.8 ± 0.3	67.6 ± 0.7	4.5 ± 0.3
Unnotched	I	240	25	3.5 ± 0.1	63.4 ± 1.1	4.34 ± 1.9
	II	240	10	3.4 ± 0.1	62.3 ± 2.3	7.1 ± 4.3
	III	210	25	3.7 ± 0.2	62.3 ± 3.0	3.4 ± 2.0
	IV	210	10	4.0 ± 0.3	64.9 ± 1.1	4.9 ± 1.1

**Table 3:** Relaxation enthalpy values for PLGA specimens determined by DSC.

PLGA specimens	Condition I	Condition II	Condition III	Condition IV
Unnotched	2.8 (±0.3) J/g	4.2 (±0.6) J/g	3.7 (±0.3) J/g	3.8 (±0.5) J/g
Notched	3.6 (±0.2) J/g	3.7 (±0.5) J/g	5.2 (±0.3) J/g	4.5 (±0.4) J/g
